# Gamma-Ray Attenuation Performance of PEEK Reinforced with Natural Pumice and Palygorskite

**DOI:** 10.3390/polym18020198

**Published:** 2026-01-11

**Authors:** Ahmed Alharbi

**Affiliations:** Department of Physics, College of Science, Qassim University, Buraydah 51452, Qassim, Saudi Arabia; ahma.alharbi@qu.edu.sa

**Keywords:** PEEK composites, palygorskite, pumice, radiation shielding, photon attenuation, sustainable materials

## Abstract

Lightweight, lead-free polymer–mineral composites have attracted increasing interest as radiation-attenuating materials for applications where reduced mass and environmental compatibility are required. In this work, the γ-ray attenuation behavior of poly(ether ether ketone) (PEEK) reinforced with natural palygorskite and pumice was evaluated at filler concentrations of 10–40 wt%. Photon interaction parameters, including the linear attenuation coefficient (μ), half-value layer (HVL), mean free path (λ), and effective atomic number ($Z_{\mathrm{eff}}$), were computed over the energy range 15 keV–15 MeV using the Phy-X/PSD platform and validated through full Geant4 Monte Carlo transmission simulations. At 15 keV, μ increased from $1.46\;{\mathrm{cm}}^{-1}$ for pure PEEK to $4.21\;{\mathrm{cm}}^{-1}$ and $8.499\;{\mathrm{cm}}^{-1}$ for the 40 wt% palygorskite- and pumice-filled composites, respectively, reducing the HVL from 0.69 cm to 0.24 cm and 0.11 cm. The corresponding $Z_{\mathrm{eff}}$ values increased from 6.5 (pure PEEK) to 9.4 (40 wt% palygorskite) and 15.3 (40 wt% pumice), reflecting the influence of higher-*Z* oxide constituents in pumice. At higher photon energies, the attenuation curves converged as Compton scattering became dominant, although pumice-filled PEEK retained marginally higher μ and shorter λ up to the MeV region. These findings demonstrate that natural mineral fillers can enhance the photon attenuation behavior of PEEK while retaining the known thermal stability and mechanical performance of the polymer matrix as reported in the literature, indicating their potential use as lightweight, secondary radiation-attenuating components in medical, industrial, and aerospace applications.

## 1. Introduction

Ionizing radiation shielding is required in medical imaging, nuclear facilities, industrial radiography, and space applications, where high-performance materials are essential to ensure the safety of operators, patients, and equipment [1,2,3]. Traditional shielding systems are dominated by high-density materials such as lead, structural steel, and Portland concrete, which offer excellent attenuation across broad photon energy ranges. However, these materials suffer from significant drawbacks, including excessive weight, limited mechanical flexibility, and serious toxicity and environmental hazards stemming from lead [4,5]. Such limitations restrict their suitability for modern applications that demand lightweight, portable, and environmentally benign alternatives  [6,7,8].

To address these challenges, polymer-based radiation shielding materials have gained increasing interest due to their low density, processability, corrosion resistance, and compatibility with additive manufacturing. Previous studies have examined polymer composites reinforced with high-*Z* fillers such as ${\mathrm{Bi}}_{2}$$O_{3}$, ${\mathrm{WO}}_{3}$, PbO, and rare-earth oxides, achieving notable improvements in photon attenuation performance [9,10,11,12]. Despite their effectiveness, the use of heavy-metal fillers introduces new issues including cost, toxicity, and recycling difficulties, which motivate the exploration of alternative filler systems for non-primary shielding applications.

In parallel, naturally occurring minerals have emerged as promising candidates for sustainable and low-cost radiation-attenuating composites. Minerals such as barite, basalt, zeolite, vermiculite, and pumice have demonstrated measurable attenuation performance when incorporated into cementitious or polymer matrices [13,14]. Among silicate-based minerals, palygorskite (attapulgite) and pumice stand out for their abundance, low cost, structural stability, and oxide-rich compositions dominated by ${\mathrm{SiO}}_{2}$ and ${\mathrm{Al}}_{2}$$O_{3}$, with minor contributions from ${\mathrm{Fe}}_{2}$$O_{3}$, MgO, CaO, $K_{2}$O, and ${\mathrm{Na}}_{2}$O. These constituents contribute to moderate effective atomic numbers ($Z_{\mathrm{eff}}\approx 8$–14), making them relevant for low- and medium-energy photon attenuation. In this work, low photon energies refer to energies below approximately 100 keV, where photoelectric absorption dominates the interaction process. Recent studies have demonstrated the attenuation potential of palygorskite-based composites [15], and pumice-containing mortars have shown measurable attenuation performance under comparable thickness conditions [16]. Nevertheless, despite their widespread use in construction, filtration, and sorption technologies, the photon interaction behavior of these minerals, particularly as fillers in high-performance polymers, remains largely unexplored.

Poly(ether ether ketone) (PEEK) is an advanced engineering polymer known for its exceptional mechanical strength, thermal stability, chemical resistance, and radiation tolerance [17]. These properties make PEEK particularly suitable for demanding aerospace, nuclear, and medical device environments. Despite the extensive literature on PEEK-based composites for structural and thermal applications, no prior study has systematically evaluated the gamma-ray attenuation behavior of PEEK reinforced with natural silicate minerals, leaving a clear research gap.

In this context, natural silicate–PEEK composites represent an environmentally friendly and lightweight class of radiation-attenuating materials that complement, rather than replace, conventional heavy-metal shielding systems. The present work investigates how mineral loading (10–40 wt%) influences the photon interaction behavior of PEEK and benchmarks the resulting attenuation performance against conventional shielding materials. It is emphasized that the objective of this study is not to propose these materials as replacements for high-*Z* primary gamma-ray shielding, but rather to quantitatively examine their attenuation behavior and identify energy ranges and application scenarios where lightweight, lead-free composites may provide useful secondary radiation attenuation. Using the Phy-X/PSD platform, photon interaction parameters, including mass and linear attenuation coefficients, half-value layer (HVL), mean free path (MFP), and effective atomic number ($Z_{\mathrm{eff}}$), are computed over the energy range 15 keV–15 MeV and validated using a dedicated Geant4 Monte Carlo transmission model [18,19,20]. Finally, the simulated performance of silicate–PEEK composites is compared with representative lead-, steel-, and concrete-based shields to identify scenarios where such materials offer an optimal balance between attenuation efficiency, mass reduction, and environmental sustainability, while leveraging the known mechanical performance of the PEEK matrix as reported in the literature.

## 2. Theory

### 2.1. Photon Attenuation in Matter

The attenuation of a monoenergetic, narrow photon beam incident on matter is governed by the exponential attenuation law [21]:

(1) $$I=I_{0}e^{-\mu x},$$

where $I_{0}$ is the incident photon intensity, *I* is the transmitted intensity after traversing a thickness *x*, and μ is the linear attenuation coefficient (LAC). The mass attenuation coefficient (MAC), defined as

(2) $$\mu_{m}=\frac{\mu }{\rho },$$

normalizes attenuation with respect to density ρ and therefore facilitates direct comparisons between materials of different densities.

A practical shielding metric derived from Equation (1) is the half-value layer (HVL), defined as the thickness required to reduce the photon intensity by 50%:

(3) $$\mathrm{HVL}=\frac{\ln2}{\mu }.$$

Materials with larger μ exhibit smaller HVL and therefore provide superior shielding efficiency, particularly in the low-energy region where the photoelectric effect dominates the interaction process. Another fundamental attenuation parameter is the mean free path (MFP), which represents the average distance traveled by a photon between successive interactions and is defined as the inverse of the linear attenuation coefficient:

(4) $$\lambda =\frac{1}{\mu }.$$

### 2.2. Composite Cross-Sections and Mixture Calculations

For compounds and heterogeneous mixtures such as the mineral-reinforced PEEK composites considered in this work, photon interaction quantities are determined using standard mixture relations. The total atomic cross-section $\sigma_{t,a}$ is computed from the elemental mass fractions $w_{i}$ and atomic weights $A_{i}$ according to [22]

(5) $$\sigma_{t,a}=\mu_{m}\;N_{A}\sum_{i}\frac{w_{i}}{A_{i}},$$

where $N_{A}$ is Avogadro’s number. The total electronic cross-section $\sigma_{t,\mathrm{el}}$ is obtained from the fractional abundance of each element and its atomic number $Z_{i}$ through mixture relations described in [23].

On this basis, two important composite metrics, the effective atomic number and effective electron density, are defined as [24,25]

(6) $$Z_{\mathrm{eff}}=\frac{\sigma_{t,a}}{\sigma_{t,\mathrm{el}}},\;N_{\mathrm{eff}}=\frac{\mu_{m}}{\sigma_{t,\mathrm{el}}}.$$

These parameters provide quantitative insights into the photon–matter interaction behavior of composite systems, especially in spectral regions influenced by elemental K-edges, where silicate-based fillers such as palygorskite or pumice exhibit characteristic changes in attenuation.

### 2.3. Mass Attenuation Coefficient of Composite Systems

For heterogeneous systems such as palygorskite–PEEK or pumice–PEEK composites, the mass attenuation coefficient is evaluated using the widely accepted mixture rule [18]:

(7) $${\left(\frac{\mu }{\rho }\right)}_{\mathrm{mix}}=\sum_{i}w_{i}{\left(\frac{\mu }{\rho }\right)}_{i},$$

where $w_{i}$ is the elemental or oxide mass fraction and ${\left(\mu /\rho \right)}_{i}$ is the MAC of the *i*th constituent. This approach is valid across all major photon interaction regimes (photoelectric absorption, Compton scattering, and pair production) and forms the basis of attenuation modeling in computational tools such as XCOM, WinXCom, and Phy-X/PSD  [19].

This formalism enables systematic evaluation of how silicate mineral phases such as Mg–Al-rich palygorskite or alkali–aluminosilicate pumice contribute to the overall attenuation efficiency of PEEK-based composites over the photon energy range 15 keV–15 MeV.

## 3. Materials

The oxide compositions of the natural palygorskite (attapulgite) and pumice used in this study were collected from published geological and radiation-shielding studies  [15,16,26,27]. These references report representative mass fractions of ${\mathrm{SiO}}_{2}$, ${\mathrm{Al}}_{2}$$O_{3}$, ${\mathrm{Fe}}_{2}$$O_{3}$, MgO, CaO, ${\mathrm{Na}}_{2}$O, and $K_{2}$O for naturally occurring silicate minerals. Table 1 summarizes the oxide fractions adopted in this work. The oxide compositions were subsequently converted to elemental mass fractions for use in both Phy-X/PSD and Geant4 simulations. Loss-on-ignition (LOI) components were excluded from the elemental conversion process, and the remaining oxide fractions were renormalized to 100 wt% prior to use as input for the Phy-X/PSD calculations and Geant4 material definitions. Because the present work was performed entirely using computational modeling, no laboratory materials or equipment were required, and supplier details are not applicable.

The computational modeling framework used poly(ether ether ketone) (PEEK) as the base polymer matrix, reinforced with either palygorskite or pumice at loadings of 10–40 wt%. Two reference systems were included for baseline comparison: pure PEEK (100 wt%) and pure mineral filler (100 wt%). Composite densities were estimated using the standard mixture rule for multiphase systems [22]:

(8) $$\frac{1}{\rho_{\mathrm{composite}}}=\sum_{i}\frac{\omega_{i}}{\rho_{i}},$$

where $\rho_{\mathrm{composite}}$ is the effective density of the mixture, $\omega_{i}$ is the weight fraction of component *i*, and $\rho_{i}$ is the density of that component. In this study, the densities used were $\rho_{\mathrm{PEEK}}=1.30\;g\;{\mathrm{cm}}^{-3}$, $\rho_{\mathrm{palygorskite}}=2.40\;g\;{\mathrm{cm}}^{-3}$, and $\rho_{\mathrm{pumice}}=1.50\;g\;{\mathrm{cm}}^{-3}$. The resulting composite densities used in the Geant4 simulations are listed in Table 2.

## 4. Methods

### 4.1. Phy-X/PSD Computations

Radiation-shielding parameters for pure PEEK and the mineral-reinforced composites were calculated using the Phy-X/PSD platform, which provides photon interaction coefficients derived from the NIST XCOM database and established mixture cross-section relations [19]. The software outputs the linear attenuation coefficient (μ), mass attenuation coefficient (μ/ρ), half-value layer (HVL), mean free path (MFP), effective atomic number ($Z_{\mathrm{eff}}$), and effective electron density ($N_{\mathrm{eff}}$) over the photon energy range from 15 keV to 15 MeV. Input data consisted of the elemental mass fractions of PEEK, palygorskite, pumice, and their composite formulations at 10, 20, 30, and 40 wt% loading, together with mixture rule densities derived in Table 2. Because Phy-X/PSD assumes homogeneous media under narrow-beam irradiation, it is well suited for analytical benchmarking prior to stochastic transport calculations and provides a consistent basis for comparing the influence of mineral loading on photon interaction probabilities.

### 4.2. Geant4 Monte Carlo Simulations

Photon transport simulations were performed using Geant4 (version 10.7.3), enabling explicit modeling of material composition, interaction physics, and detector configuration [20]. The electromagnetic physics list G4EmStandardPhysics_option4 was selected to ensure accurate treatment of low- and intermediate-energy photon processes, including photoelectric absorption, Rayleigh scattering, Compton scattering, pair production, and secondary-electron transport.

**Figure 1:**
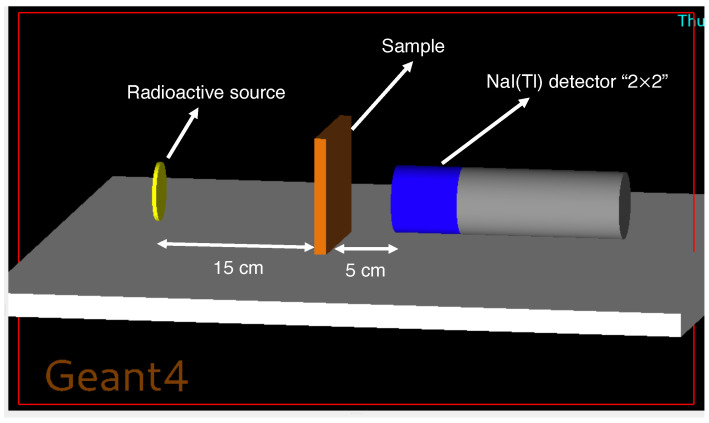
Geant4 narrow-beam transmission geometry used to determine the linear attenuation coefficient. A monoenergetic γ-ray source (yellow) irradiates the PEEK–mineral composite slab (orange), and a NaI(Tl) detector ($2^{\prime \prime }\times$ $2^{\prime \prime }$, blue/gray) positioned downstream records the transmitted photon spectrum. The source–sample and sample–detector distances are fixed at 15 cm and 5 cm, respectively, for all materials and energies. This geometry enables the direct comparison between Monte Carlo and analytical attenuation results.

A narrow-beam transmission geometry was implemented in which a monoenergetic γ-ray source (15 keV–15 MeV) irradiated a planar slab of material, and a NaI(Tl) detector placed behind the sample recorded the transmitted photons. A uniform sample thickness of x=1.0 cm was used for all materials and photon energies. The source–sample and sample–detector distances were fixed at 15 cm and 5 cm, respectively, for all compositions to ensure direct comparability. For all simulations, the sample density and elemental composition were assigned according to the mixture definitions in Table 2. The Geant4 attenuation geometry used in this work is illustrated in Figure 1.

Each simulation was executed with $1\times 10^{6}$ primary photons, yielding statistical uncertainties below 1–2% in the computed transmission values and derived attenuation coefficients. The linear attenuation coefficient μ for each material and energy was obtained using the logarithmic transmission expression

$$\mu =-\frac{1}{x}\;\ln\left(\frac{I}{I_{0}}\right),$$

where $I_{0}$ is the number of incident photons and *I* is the number recorded by the NaI(Tl) detector after traversing a thickness *x* of the sample. To preserve narrow-beam conditions, the counting was restricted to photons registered in the forward direction, thereby minimizing the influence of large-angle scattered contributions. The remaining differences between the Geant4 and Phy-X/PSD results were within ±2.5%, indicating excellent quantitative agreement between the two approaches and confirming the internal consistency of the material definitions and transport-physics models.

## 5. Results and Discussion

This section presents the photon attenuation behavior of PEEK-based composites reinforced with palygorskite and pumice at loading levels of 0, 10, 20, 30, and 40 wt%. The evaluated photon attenuation parameters include the linear attenuation coefficient (μ), mass attenuation coefficient (μ/ρ), half-value layer (HVL), mean free path (λ), and effective atomic number ($Z_{\mathrm{eff}}$), calculated over photon energies ranging from 15 keV to 15 MeV using the Phy-X/PSD computational tool. Full Geant4 Monte Carlo transmission simulations were performed for all compositions to validate the Phy-X/PSD results and ensure consistency between analytical and stochastic approaches. The resulting attenuation trends follow well-defined physical behavior and demonstrate consistent enhancement with increasing mineral filler content. In the present discussion, low photon energies refer to energies below approximately 100 keV, where photoelectric absorption dominates the interaction process.

### 5.1. Gamma-Ray Attenuation Behavior of PEEK–Palygorskite Composites

**Figure 2:**
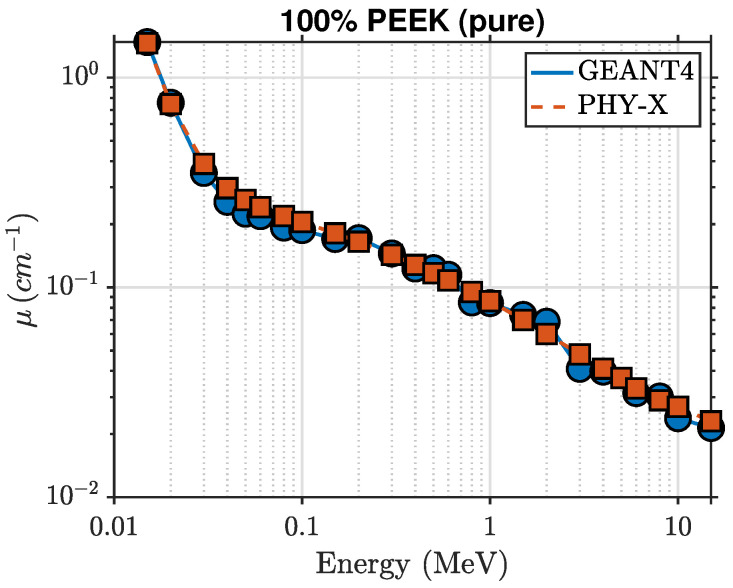
Photon attenuation comparison for 100% PEEK obtained from GEANT4 and Phy-X/PSD.

**Figure 3:**
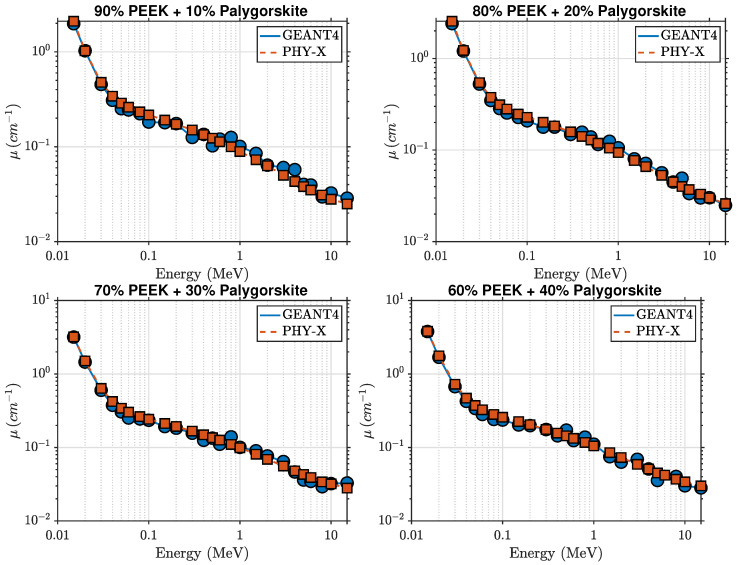
Comparison of GEANT4 and Phy-X/PSD attenuation coefficients for PEEK–palygorskite composites at 10, 20, 30, and 40 wt%.

The photon attenuation behavior of pure PEEK and the PEEK–palygorskite composites is shown in Figure 2 and Figure 3. The linear attenuation coefficient (μ) decreases with increasing photon energy for all compositions, reflecting the expected transition from photoelectric absorption at low energies to Compton scattering in the intermediate-energy region. At 15 keV, μ increases from approximately $1.457\;{\mathrm{cm}}^{-1}$ for pure PEEK to $3.808\;{\mathrm{cm}}^{-1}$ for the 40 wt% composite, indicating a strong enhancement in low-energy photon absorption resulting from the increased mineral content. Intermediate loadings (10–30 wt%) follow a smooth monotonic increase, yielding μ=2.102, 2.551, and $3.206\;{\mathrm{cm}}^{-1}$ for 10, 20, and 30 wt% palygorskite, respectively.

Above about 0.1 MeV, μ decreases rapidly for all materials and the attenuation curves begin to converge as Compton scattering becomes the dominant interaction mechanism. Nevertheless, small composition-dependent differences persist. At 0.1 MeV, pure PEEK exhibits $\mu \approx 0.205\;{\mathrm{cm}}^{-1}$, increasing to $\mu \approx 0.258\;{\mathrm{cm}}^{-1}$ for the 40 wt% composite. At 1 MeV, μ increases from $0.086\;{\mathrm{cm}}^{-1}$ (pure PEEK) to $0.105\;{\mathrm{cm}}^{-1}$ (40 wt%), and remains slightly higher across the full MeV range considered.

The agreement between GEANT4 simulations and Phy-X/PSD calculations is excellent across all photon energies, with deviations below 2.5%. This confirms that the implemented material definitions, elemental mass fractions, and electromagnetic cross-section models are consistent across both computational approaches.

**Figure 4:**
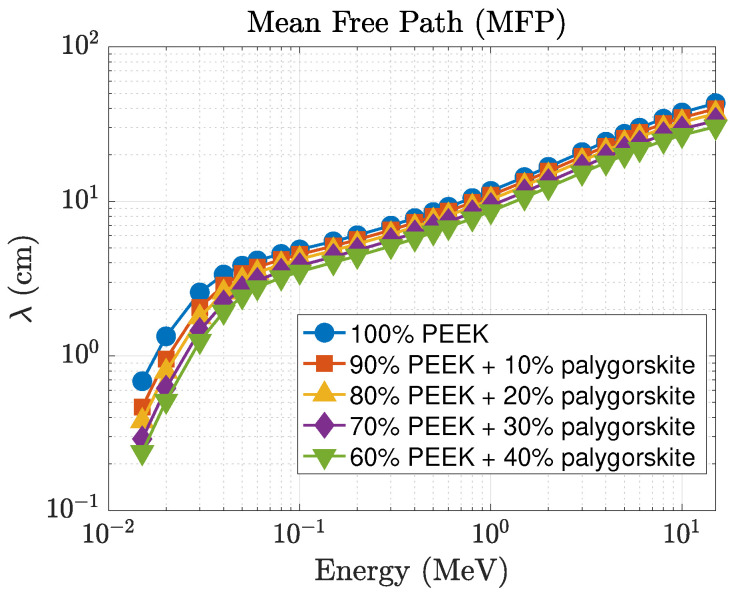
Energy dependence of the mean free path (λ) for PEEK–palygorskite composites.

**Figure 5:**
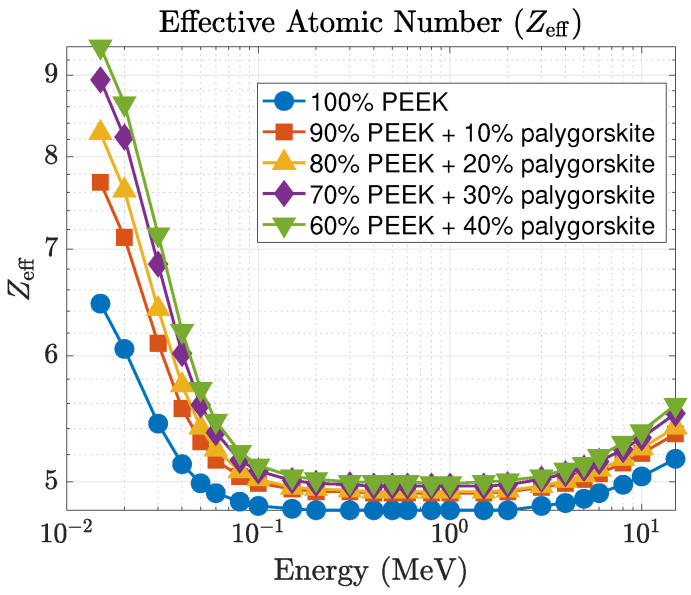
Effective atomic number ($Z_{\mathrm{eff}}$) variation with photon energy for PEEK–palygorskite composites.

The enhancement in attenuation is clearly reflected in the mean free path (λ), shown in Figure 4. At 15 keV, λ decreases from 0.686 cm for pure PEEK to 0.263 cm for the 40 wt% composite, representing a nearly three-fold improvement in photon stopping capability. At 0.1 MeV, λ decreases from 4.885 cm (pure PEEK) to 3.880 cm (40 wt%), and at 1 MeV, it decreases from 11.692 cm to 9.538 cm. These reductions correspond to enhanced photon attenuation and shorter penetration depths. The effective atomic number ($Z_{\mathrm{eff}}$), displayed in Figure 5, also shows a strong dependence on filler concentration at low energies. At 15 keV, $Z_{\mathrm{eff}}$ increases from 6.47 (pure PEEK) to 9.38 for the 40 wt% composite, while intermediate loadings produce $Z_{\mathrm{eff}}=7.71$, 8.28, and 8.94 for the 10, 20, and 30 wt% composites, respectively. This behavior is consistent with the strong $Z^{3}$–$Z^{4}$ dependence of the photoelectric effect. As the energy increases beyond ∼ 0.3 MeV, all curves gradually converge toward $Z_{\mathrm{eff}}\approx 5$, consistent with Compton-dominated interaction regimes.

### 5.2. Gamma-Ray Attenuation Behavior of PEEK–Pumice Composites

**Figure 6:**
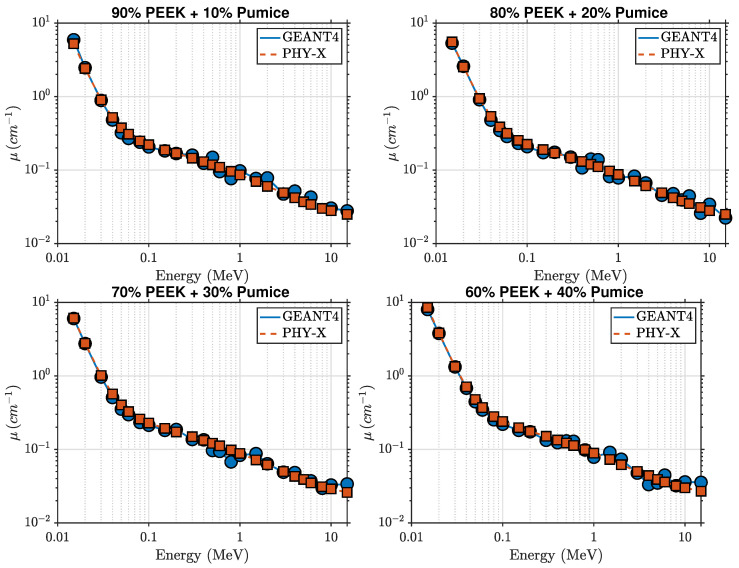
Comparison of GEANT4 and Phy-X/PSD attenuation coefficients for PEEK–pumice composites at 10, 20, 30, and 40 wt%.

Figure 2 and Figure 6 show the linear attenuation coefficients for pure PEEK and PEEK–pumice composites over the photon energy range 15 keV–15 MeV. As with the palygorskite system, the linear attenuation coefficient (μ) decreases monotonically with increasing photon energy, reflecting the transition from photoelectric absorption at low energies to Compton scattering at intermediate energies. However, the pumice-filled composites exhibit substantially stronger attenuation at low energies. At 15 keV, μ increases from $1.457\;{\mathrm{cm}}^{-1}$ for pure PEEK to approximately $8.499\;{\mathrm{cm}}^{-1}$ for the 40 wt% pumice composite, representing more than double the enhancement observed for the palygorskite-loaded materials.

Intermediate filler loadings (10, 20, and 30 wt%) follow a smooth, progressive trend, with μ values of 2.414, 2.539, and $2.779\;{\mathrm{cm}}^{-1}$ at 15 keV, respectively. This confirms that the attenuation improvement scales consistently with pumice content. The superior low-energy performance is primarily attributed to the higher concentrations of Fe, Ca, K, and Na present in pumice, which elevate the effective atomic number and strengthen the photoelectric effect, whose cross-section depends strongly on $Z^{3}$–$Z^{4}$ in the sub-50 keV region.

Beyond approximately 0.1 MeV, the attenuation curves for all compositions begin to converge as Compton scattering becomes dominant and the dependence on atomic number decreases. At 1 MeV, pure PEEK shows $\mu \approx 0.086\;{\mathrm{cm}}^{-1}$, whereas the 40 wt% pumice composite reaches $\mu \approx 0.089\;{\mathrm{cm}}^{-1}$, retaining a small but consistent enhancement. Even above 10 MeV, where pair production begins to contribute, the overall ordering of the curves remains physically consistent.

**Figure 7:**
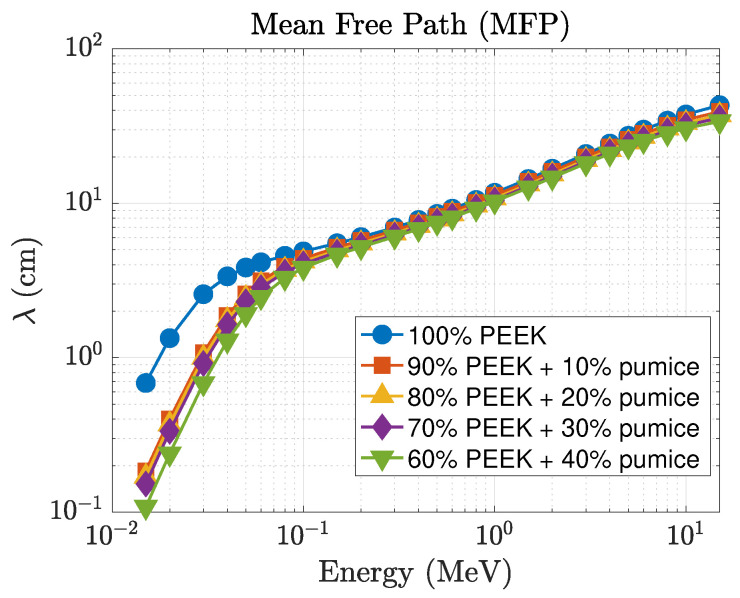
Energy dependence of the mean free path (λ) for PEEK–pumice composites.

**Figure 8:**
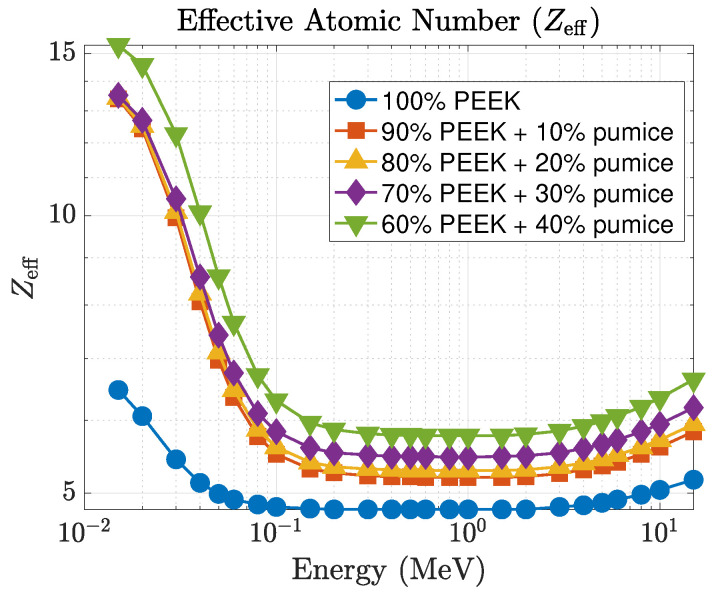
Effective atomic number ($Z_{\mathrm{eff}}$) as a function of photon energy for PEEK–pumice composites.

The mean free path (λ), shown in Figure 7, exhibits the expected inverse relationship with μ. At 15 keV, λ decreases from 0.686 cm for pure PEEK to 0.118 cm for the 40 wt% composite, which represents more than a five-fold reduction in photon penetration depth. At 0.1 MeV, the reduction is from 4.885 cm to 4.172 cm, and at 1 MeV from 11.692 cm to 11.219 cm. Although the differences are smaller in the MeV region, the pumice-filled composites consistently maintain shorter penetration depths than neat PEEK across the full energy spectrum. The effective atomic number ($Z_{\mathrm{eff}}$), presented in Figure 8, further highlights the superior low-energy behavior. At 15 keV, $Z_{\mathrm{eff}}$ increases from 6.47 (pure PEEK) to 15.31 for the 40 wt% pumice composite, reflecting the influence of higher-*Z* constituents in natural pumice. Intermediate loadings produce $Z_{\mathrm{eff}}=12.43$, 12.52, and 12.69 for the 10, 20, and 30 wt% composites, respectively. Even in the 40–100 keV range where $Z_{\mathrm{eff}}$ gradually decreases for all materials, the pumice-filled composites retain higher effective atomic numbers than the palygorskite series. As the photon energy exceeds ∼0.3 MeV, all $Z_{\mathrm{eff}}$ curves converge toward values between 5 and 6, reflecting the dominance of Compton scattering.

The Phy-X/PSD results for the pumice series are smooth and physically consistent, with no anomalous crossings. These results demonstrate clearly that mineral composition, particularly the presence of higher-*Z* oxides, has a dominant influence on the shielding performance of PEEK-based composites. Pumice-reinforced PEEK therefore represents a promising candidate for lightweight, secondary radiation-attenuating components in low- and medium-energy photon environments, where enhanced photoelectric absorption and reduced penetration depth are beneficial but primary high-*Z* shielding is not required.

### 5.3. Practical Considerations for Composite Fabrication

In translating the simulated PEEK–mineral systems into practical shielding components, several processing and materials engineering considerations must be addressed. A primary requirement is the uniform dispersion of palygorskite or pumice particles within the PEEK matrix. Poor dispersion or particle agglomeration can introduce stress concentrators, reduce mechanical integrity, and alter local density, thereby affecting both structural performance and the predictability of photon attenuation. Previous work on mineral-filled thermoplastics demonstrates that micron-scale silicate powders can be blended homogeneously at filler contents up to 30–40 wt% using melt compounding or twin-screw extrusion when appropriate temperature profiles and screw configurations are employed [15,16,17]. These reported processing windows guided the assumed loading limits used in the present modeling study.

At filler levels approaching 40 wt%, melt viscosity increases substantially. Because PEEK must already be processed at elevated temperatures (360–400 °C) to ensure complete melting and adequate flow, the addition of rigid silicate or aluminosilicate particles further restricts chain mobility, potentially requiring higher injection pressures, extended residence times, or the use of processing aids to maintain manufacturability [17]. Good interfacial adhesion between PEEK and the mineral phase is likewise essential. Surface treatments or coupling agents may reduce interfacial voids and enhance load transfer, contributing to improved mechanical stability and more uniform density across the molded part.

High inorganic contents also lead to characteristic changes in bulk mechanical properties. As commonly observed in mineral-reinforced polymers, stiffness and dimensional stability typically increase, while ductility and impact resistance decrease once the filler content exceeds 20–30 wt% [13,16]. For this reason, the PEEK–palygorskite and PEEK–pumice formulations considered in this work are treated primarily as model systems for benchmarking photon attenuation, rather than as fully optimized structural composites. For long-term, load-bearing shielding applications, similar mineral formulations could be incorporated into high-modulus grades of PEEK or related polyaryletherketones designed specifically for mechanical robustness under cyclic stresses, thermal cycling, and ionizing radiation exposure [17].

Additional factors affecting manufacturability include particle size distribution, porosity, and moisture content. Palygorskite contains structural water and surface hydroxyl groups that must be removed through controlled pre-drying to prevent steam formation or voids during the melt processing. Pumice, owing to its highly porous morphology, can trap air and moisture; inadequate drying or degassing can cause internal porosity, density variations, and inconsistent attenuation behavior in the final part. Proper powder drying, extruder venting, and mold design are therefore essential to achieving reproducible density and consequently, reproducible radiation-shielding performance in fabricated PEEK–mineral composite components.

### 5.4. Comparative Assessment of PEEK–Palygorskite and PEEK–Pumice Systems

The simulation results show that both PEEK–palygorskite and PEEK–pumice composites exhibit a systematic improvement in photon attenuation with increasing mineral content. In both systems, the linear attenuation coefficient increases, the mean free path decreases, and the effective atomic number rises as the filler concentration is increased. These trends reflect the combined effects of the embedded oxides, which enhance photoelectric absorption and Compton scattering probabilities, especially at low and intermediate photon energies [10,11,12].

A direct comparison reveals that the PEEK–pumice system provides noticeably stronger low-energy attenuation than its palygorskite counterpart. At photon energies below approximately 100 keV, the PEEK–pumice composites consistently exhibit larger values of μ and $Z_{\mathrm{eff}}$, owing to the higher concentrations of ${\mathrm{Fe}}_{2}$$O_{3}$, CaO, $K_{2}$O, and ${\mathrm{Na}}_{2}$O present in pumice. These oxides possess higher atomic numbers than the Mg/Al-rich aluminosilicate structure of palygorskite, leading to more pronounced photoelectric absorption, which scales approximately as $Z^{3}$–$Z^{4}$ in this energy region. Consequently, PEEK–pumice formulations are particularly well suited for shielding applications involving diagnostic X-rays or low-energy gamma emitters where enhanced photoelectric interactions are desirable.

PEEK–palygorskite composites, while yielding somewhat lower photon attenuation, offer alternative advantages that may be relevant in practical applications. The fibrous, high-aspect-ratio morphology of palygorskite can improve dimensional stability, creep resistance, and dispersion within the polymer matrix, potentially facilitating more manageable processing at elevated filler contents compared with granular pumice powders [15]. Moreover, the lower iron content of palygorskite may be beneficial in environments where activation concerns or magnetic compatibility are important design considerations.

At intermediate and high photon energies (above approximately 0.3–1 MeV), the attenuation curves for all compositions converge. In this regime, the photon interaction mechanisms transition toward Compton scattering and pair production, both of which exhibit reduced sensitivity to moderate differences in effective atomic number [18,21]. Density therefore becomes the dominant factor governing attenuation, and the performance difference between the two composite systems becomes less pronounced.

Selection between palygorskite- and pumice-reinforced PEEK should thus be guided by the target photon energy range, along with practical considerations such as mechanical requirements, processing constraints, cost, and long-term material stability. Pumice fillers offer superior low-energy attenuation, whereas palygorskite may provide better manufacturability and enhanced mechanical stability at comparable loading levels.

### 5.5. Comparison with Conventional Shielding Materials

To contextualize the photon attenuation performance of the PEEK–palygorskite and PEEK–pumice systems, their behavior at 662 keV was compared with representative values for established shielding materials such as lead, structural steel, and ordinary Portland concrete. Reference values were obtained from NIST XCOM data and previously reported measurements [14,21,22,23,25]. Table 3 summarizes the linear attenuation coefficients and half-value layers (HVLs) for these materials, together with the 40 wt% PEEK-based composites investigated in this study.

As expected, lead exhibits the highest attenuation ($\mu \approx 1.24\;{\mathrm{cm}}^{-1}$), followed by structural steel ($\mu \approx 0.51\;{\mathrm{cm}}^{-1}$) and ordinary Portland concrete ($\mu \approx 0.23\;{\mathrm{cm}}^{-1}$). In comparison, the simulated PEEK–palygorskite and PEEK–pumice composites yield $\mu =0.176\;{\mathrm{cm}}^{-1}$ and $0.151\;{\mathrm{cm}}^{-1}$, respectively, which fall within the range reported for lightweight concretes, polymer–cement mixtures, and mineral-filled polymer systems [13,15,16]. Their corresponding HVLs of 3.9–4.6 cm demonstrate practical photon attenuation capability while maintaining advantages in density, chemical stability, and manufacturability.

This comparison reinforces that PEEK-based mineral composites are not intended to supersede high-density metallic shields in primary radiation barriers, but they do offer a valuable balance between attenuation performance, mass efficiency, lead-free composition, and structural robustness. As such, these composites represent promising candidates for secondary shielding structures, lightweight housings, portable or modular barriers, and radiation-tolerant components in medical, laboratory, nuclear, and aerospace applications, where reduced weight and mechanical durability are required, rather than for primary radiation barriers.

Lead equivalence is a thickness- and energy-dependent quantity commonly used in diagnostic X-ray shielding design. Because the present work focuses on fundamental photon attenuation parameters over a broad energy range (15 keV–15 MeV), lead equivalence values were not explicitly tabulated. However, the reported linear attenuation coefficients and half-value layers enable the direct calculation of lead equivalence at specific diagnostic X-ray energies for a given material thickness. It is emphasized that the studied PEEK–mineral composites are not intended to replace lead-based barriers in regulated medical installations, but rather to provide lightweight, lead-free secondary attenuation in non-primary shielding applications.

### 5.6. Radiation-Induced Aging Considerations

Radiation-induced aging is an important factor governing the long-term performance of polymer–mineral composites in practical radiation environments. Although the present study focuses on photon attenuation behavior, it should be noted that ionizing radiation can induce structural, optical, and chemical changes in both polymer matrices and inorganic fillers.

For oxide-based materials, previous studies have reported radiation-induced defect formation, color-center generation, and changes in optical properties in ${\mathrm{SiO}}_{2}$-based systems under gamma and charged-particle irradiation [28]. Similar degradation effects have been observed in MgO ceramics exposed to heavy-ion irradiation, including defect clustering and the evolution of radiation-induced optical absorption bands [29]. In alumina and related oxide systems, cathodoluminescence studies have demonstrated partial recovery of radiation damage through defect annealing and recombination processes, highlighting the dynamic nature of radiation-induced defects in ceramic materials [30].

For high-performance polymers such as polyetheretherketone (PEEK), radiation exposure is known to induce competing crosslinking and chain-scission mechanisms, depending on absorbed dose, dose rate, and irradiation environment. These processes can influence mechanical integrity, thermal stability, and long-term durability. Nevertheless, PEEK has been widely reported to exhibit superior radiation tolerance compared with many commodity polymers, supporting its use in radiation-tolerant structural and functional applications [17].

A comprehensive evaluation of radiation-induced aging effects, including dose-dependent mechanical degradation, microstructural evolution, and combined radiation–thermal aging, is beyond the scope of the present work. Such effects will be addressed in future experimental and simulation-based studies aimed at assessing the long-term performance of PEEK–mineral composites under realistic radiation exposure conditions.

## 6. Conclusions

This work evaluated the photon attenuation characteristics of PEEK-based composites reinforced with palygorskite and pumice over 15 keV–15 MeV using Phy-X/PSD calculations, validated by full GEANT4 Monte Carlo transmission simulations. Both minerals enhanced the photon attenuation performance of PEEK, with the linear attenuation coefficient (μ) decreasing and the mean free path (λ) increasing with increasing photon energy, as photon interactions transitioned from photoelectric absorption to Compton scattering and pair production. Increasing the filler content up to 40 wt% raised the composite density and effective atomic number, leading to substantial reductions in the half-value layer. At 15 keV, the 40 wt% palygorskite composite increased μ from 1.46 to $4.21\;{\mathrm{cm}}^{-1}$, while pumice produced a stronger enhancement to $8.50\;{\mathrm{cm}}^{-1}$, accompanied by a reduction in λ to approximately 0.11 cm. The effective atomic number rose to 9.38 for palygorskite and 15.31 for pumice at low photon energies, confirming the influence of higher-*Z* oxides. Although differences between compositions become smaller above the Compton-dominated energy region, both systems maintained consistently improved attenuation compared with pure PEEK. PEEK–pumice composites demonstrated the strongest photon attenuation across low and medium energies, whereas PEEK–palygorskite systems offer potential processing advantages due to the fibrous nature of the filler.

The investigated PEEK–mineral composites are not intended to replace lead-based primary radiation barriers. Instead, they are designed to serve as lightweight, lead-free secondary attenuation materials for applications such as wall and panel linings in laboratories adjacent to X-ray rooms, housings for medical and nuclear instrumentation, portable protective components, and building elements in environments with elevated natural radiation levels. The findings indicate that PEEK–mineral composites are promising candidates for lightweight, lead-free secondary radiation-attenuating components in medical imaging, nuclear instrumentation, aerospace structures, and portable protective systems, where efficient attenuation must be balanced with low mass and mechanical robustness.

## Figures and Tables

**Table 1:** Representative oxide composition (wt%) of natural palygorskite and pumice used in this study, based on Refs. [15,16,26,27].

Oxide	Palygorskite (wt%)	Pumice (wt%)
${\mathrm{SiO}}_{2}$	55.0	63.4
${\mathrm{Al}}_{2}$ $O_{3}$	10.2	18.1
${\mathrm{Fe}}_{2}$ $O_{3}$	1.5	3.2
MgO	6.1	1.0
CaO	2.0	2.5
${\mathrm{Na}}_{2}$O	0.6	4.1
$K_{2}$O	0.7	4.0
Loss-on-Ignition (LOI)	24.0	3.7

**Table 2:** Densities of PEEK–palygorskite and PEEK–pumice composites at different filler loadings (10–40 wt%), calculated using Equation (8).

Composite	PEEK (wt%)	Filler (wt%)	Density (g ${\mathrm{cm}}^{-3}$)
PEEK–Palygorskite Composites			
PEEK (100 wt%)	100	0	1.30
PEEK + 10 wt% palygorskite	90	10	1.362
PEEK + 20 wt% palygorskite	80	20	1.431
PEEK + 30 wt% palygorskite	70	30	1.507
PEEK + 40 wt% palygorskite	60	40	1.592
PEEK–Pumice Composites			
PEEK (100 wt%)	100	0	1.30
PEEK + 10 wt% pumice	90	10	1.318
PEEK + 20 wt% pumice	80	20	1.336
PEEK + 30 wt% pumice	70	30	1.354
PEEK + 40 wt% pumice	60	40	1.373

**Table 3:** Representative linear attenuation coefficients and half-value layers (HVLs) at 662 keV for conventional shielding materials and the simulated PEEK-based composites (40 wt% filler).

Material	μ (${\mathrm{cm}}^{-1}$)	HVL (cm)
Lead	1.24	0.56
Structural steel	0.51	1.36
Ordinary Portland concrete	0.23	3.01
PEEK–palygorskite (40 wt%)	0.176	3.93
PEEK–pumice (40 wt%)	0.151	4.59

## Data Availability

The original contributions presented in this study are included in the article. Further inquiries can be directed to the corresponding author.
